# Direct comparison of 3D and 2D cultivation reveals higher osteogenic capacity of elderly osteoblasts in 3D

**DOI:** 10.1186/s13018-020-02153-z

**Published:** 2021-01-06

**Authors:** Stephan Payr, Elizabeth Rosado-Balmayor, Thomas Tiefenboeck, Tim Schuseil, Marina Unger, Claudine Seeliger, Martijn van Griensven

**Affiliations:** 1 Department of Experimental Trauma Surgery, Klinikum Rechts der Isar, Technical University Munich, Munich, Germany; 2 Department of Orthopedics and Trauma Surgery, Division of Trauma Surgery, Medical University of Vienna, Waehringer Guertel 18-20, 1090 Vienna, Austria; 3 Department IBE, MERLN Institute, Maastricht University, Universiteitssingel 40, 6229 ER Maastricht, The Netherlands; 4 Department cBITE, MERLN Institute, Maastricht University, Universiteitssingel 40, 6229 ER Maastricht, The Netherlands

**Keywords:** Donor age, Osteoblasts, 2D/3D cultivation

## Abstract

**Background:**

The aim of this study was the investigation of the osteogenic potential of human osteoblasts of advanced donor age in 2D and 3D culture.

**Methods:**

Osteoblasts were induced to osteogenic differentiation and cultivated, using the same polystyrene material in 2D and 3D culture for 2 weeks. Samples were taken to evaluate alkaline phosphatase (ALP) activity, mineralization and gene expression.

**Results:**

Osteoprotegerin (OPG) levels were significantly increased (8.2-fold) on day 7 in 3D compared to day 0 (*p* < 0.0001) and 11.6-fold higher in 3D than in 2D (*p* < 0.0001). Both culture systems showed reduced osteocalcin (OC) levels (2D 85% and 3D 50% of basic value). Collagen type 1 (Col1) expression was elevated in 3D on day 7 (1.4-fold; *p* = 0.009). Osteopontin (OP) expression showed 6.5-fold higher levels on day 7 (*p* = 0.002) in 3D than in 2D. Mineralization was significantly higher in 3D on day 14 (*p* = 0.0002).

**Conclusion:**

Advanced donor age human primary osteoblasts reveal significantly higher gene expression levels of OPG, Col1 and OP in 3D than in monolayer. Therefore, it seems that a relatively high potential of bone formation in a natural 3D arrangement is presumably still present in osteoblasts of elderly people.

**Trial registration:**

5217/11 on the 22nd of Dec. 2011.

## Introduction

Trauma of the elderly is a rising issue in trauma and orthopaedic surgery. Fracture healing itself is a complex physiologic process in which numerous cytokines, angiogenic factors, proteases and morphogens with significant roles are involved [1, 2]. Despite intensive research, many regulation mechanisms involved in bone healing are still not clear [3]. One cell type in the centre of attention are the osteoblasts, which are fibroblast-like cells derived from pluripotent mesenchymal stem cells (MSCs) [4]. Osteoblasts are bone-forming cells and express typical osteoblastic markers such as collagen type 1 (Col1), osteocalcin (OC), alkaline phosphatase (ALP) and osteoprotegerin (OPG) [5–7]. These osteoblastic markers indicate osteoblast’s activity and represent bone formation, except for OPG, which is a glycoprotein produced by osteoblasts suppressing bone resorption and increases bone mass [8].

Human aging is associated with bone loss leading to bone fragility and increased risk of fractures and morbidity in the geriatric population leading to a decline in the quality of life for the elderly as well as a substantial burden on the health care system [9]. Age-related osteoblast dysfunction is the main cause of age-related bone loss in both men and women beyond the fifth decade [10]. In general terms, age-related bone loss is characterized by reduced bone formation due to decreased number of osteoblasts and lowered activity. A further cellular mechanism for bone loss is an impairment of the osteogenic potential because of decreased MSCs leading to reduced expression of osteoblastic markers [10, 11]. Age-related osteoblast dysfunction is also associated with impaired proliferation, a decline in functional lifespan and a decrease in differentiation, function and activity [10]. These age-related changes are caused by extrinsic mechanisms mediated by hormones and growth factors and intrinsic mechanisms caused by osteoblastic cell senescence [12]. New approaches to avoid cell senescence are the addition of growth factors and the usage of three-dimensional cultivation [13, 14]. Several growth factors that enhance osteoblast activity and consequently bone formation include insulin-like growth factors (IGFs) [15, 16], bone morphogenetic proteins (BMPs) [17] and prostaglandins [18]. Using a 3D cultivation system is supposed to mimic physiological conditions and provide a more accurate in vitro representation of in vivo conditions for cells undergoing osteogenic differentiation compared to monolayer cultivation resulting in different cell-cell contact and interaction between cells [19]. Although studies using monolayer culture are easy and good, 3D culture systems are necessary to reproduce in vivo conditions and to mimic the complexity of tissue. 3D cultures have a high biological relevance for 3D tissues and are important for better understanding of human tissue physiology. In the literature, 3D cultivation leads to improved proliferation and differentiation in cell culture [14, 19–21].

The aim of this study was the investigation of the osteogenic potential of primary human osteoblasts of advanced age in 2D compared to 3D cultivation systems to identify mechanisms for impaired fracture healing in elderly patients in further studies. This insight might be important to prevent and manage healing complications in the elderly in the future.

## Methods

### Cell isolation and cultivation

Human primary osteoblasts were isolated from femoral heads of 6 female patients (age 75.25 ± 7.6) scheduled to undergo hip replacement surgery. Informed consent was obtained from all participants. The study was conducted according to the Declaration of Helsinki in its latest amendment. Cells were isolated and cultured in monolayer according to our standard operation procedure, as previously described [22]. Briefly, the cancellous bone was extracted and washed three times. The bone fragments were placed in flasks and osteogenic culture medium—10% fetal calf serum (Sigma-Aldrich, Munich, Germany), 1% penicillin/streptomycin (PAA, Pasching, Austria), 50 μg/ml L-Ascorbat-2-Phophat (Sigma-Aldrich, Munich, Germany) in Dulbecco’s modified Eagle’s medium (DMEM) low glucose (Sigma-Aldrich, Munich Germany)—was added. The bone fragments were incubated for 7 days at 37 °C. Then, the medium was changed and the cells were again incubated for one more week. From then on, the medium was changed twice a week. Bone fragments were removed and osteoblasts were proliferated in monolayer culture in T75 culture flasks until passage 3 and then brought into 2D or 3D culture. For monolayer cultivation, 1 × 10^4^ cells/cm^2^ were seeded in 24-well plates. Cells were cultured in osteogenic culture medium— 5% FCS (Sigma-Aldrich, Munich, Germany), 1% pen/strep (PAA, Pasching, Austria), 10 mM β-glycerolphosphate (Sigma-Aldrich, Munich, Germany), 1.56 mM CaCl_2_ (Sigma-Aldrich, Munich, Germany), 100 nM dexamethasone (Sigma-Aldrich, Munich, Germany), 0.025 M HEPES (PAA, Pasching, Austria), 0.2 mM L-ascorbic-2-phosphat (Sigma-Aldrich, Munich, Germany) in DMEM low glucose (Sigma-Aldrich, Munich, Germany). A 200-μm-thick polystyrene scaffold (Alvatex Scaffolds, Reinnervate, Sedgefield, England) was used as a scaffold (∅ 22 mm) for 3D culture. The scaffold characteristics are 13 μm thin linings and lacunae with a mean diameter of 40 μm. Osteoblasts were seeded at 1.5 × 10^6^ cells per scaffold after the scaffolds were rinsed with 70% ethanol and washed with culture medium. Scaffolds with seeded cells were incubated for 2 h. After incubation, scaffolds were put in 6-well plates with 8 ml culture medium as described above. Medium was changed every second day. For evaluating 3D culture, scaffolds were divided to obtain samples for each investigation. For evaluating 2D culture, cells were pooled from two wells out of a 24-well plate for each investigation. Samples were collected on days 0, 3, 7 and 14 to perform analysis of alkaline phosphatase (ALP) activity, alizarin red staining (mineralization), sulforhodamine B determination (SRB) and gene expression with reverse transcriptase PCR (RT-qPCR).

### Alkaline phosphatase activity

Cells and scaffold samples at the abovementioned times were transferred in alkaline phosphate substrate solution containing of 3.5 mM 4-nitrophenyl-phosphate-di-sodium-hexahydrit salt (Sigma-Aldrich) in a 0.1 mM ALP buffer (50 mM glycine, 100 mM tris-base, 2 mM MgCl_2_, pH 10.5 – Sigma-Aldrich) and incubated at 37 °C for 30 min. As negative control, ALP substrate solution without cells was used. Absorbance was measured at 405 nm, and p-nitrophenol concentrations were calculated according to standard curve and divided through the protein content calculated with SRB determination [23].

### Alizarin red staining (mineralization)

Alizarin red staining and measurement were performed to measure mineralization. Cultured cells were fixed with ice-cold ethanol 96% for 30 min in a 24-well plate. After washing twice with dH_2_O, 400 μl alizarin red staining solution was added in each well for 10 min, washed again four times. The stain was eluted for 20 min into a solution of 10% hexadecylpyridiniumchloride (Sigma-Aldrich). Absorbance was read at 562 nm. Alizarin red concentration for samples was calculated according to standard curve and results were normalized to the protein content analysed via SRB staining [23].

### Sulforhodamine B determination (SRB)

As mentioned above SRB staining was performed to normalize alizarin red staining and ALP activity. Cells were fixed with methanol for 15 min at room temperature after culture medium was removed and cells washed with DPBS. Next, the SRB staining solution was added and incubated for 30 min at room temperature on a shaker. After removing SRB staining solution and washing the plates with 1% acetic acid solution followed by 10 mM unbuffered TRIS solution, cells were incubated again at room temperature on the shaker for 15 min. Absorbance was read at 565 and 690 nm. Total protein in osteoblasts was calculated using the following equation: Total protein [μg/μl] = (OD-0.036)/0.036 [24].

### Total RNA isolation and reverse transcriptase-polymerase chain reaction

Cell-scaffold composites and cells in 2D of each group were taken and lysed using TRIzol reagent (Sigma-Aldrich, Munich, Germany) according to our standard operation procedure [25, 26]. The purity and concentration of total RNA was determined by spectrophotometry (Biophotometer + HELLMA tray cell, Eppendorf, Hamburg, Germany). cDNA synthesis [27] was performed using first-strand cDNA synthesis kit (Fermentas, St. Leon-Rot, Germany) according to manufacturer’s instruction. Reverse transcriptase polymerase chain reaction (RT-PCR) amplification was performed and monitored using a Master Cycler S (Eppendorf, Hamburg, Germany).

The thermal cycling conditions comprised the initial steps at 98 °C for 3 min. The amplification step of the cDNA products was performed with 40 PCR cycles, consisting of a denaturation step at 98 °C for 10 s and an annealing step at a variable temperature depending on the primer for 10 s. As internal control, β-tubulin was chosen as the housekeeping gene. All cDNA samples (30 ng of cDNA) were analysed in duplicates. Quantitative polymerase chain reaction (q-PCR) amplification was performed and monitored using a Q-PCR CFX96 (BioRad, California, USA). Relative quantification of gene expression was performed using the comparative ΔΔCt method. The following genes were used for analysis: alkaline phosphatase (ALP), osteoprotegerin (OPG), osteocalcin (OC), osteopontin (OP) and collagen type 1α1 (Col1). Amplification primers are listed in Table 1. Gene expression data were reported as the mean ± standard deviation of the real-time PCR analyses. For statistical analysis ΔΔCt values were used.

**Table 1:** Human primers used for the quantitative PCR evaluation

Gene	Forward primer 5ʼ ➔ 3ʼ	Reverse primer 5ʼ ➔ 3ʼ	Accession number
**OC**	CCAGCGGTGCAGAGTCCAGC	GACACCCTAGACCGGGCCGT	NM-199173.3
**OP**	CTCCATTGACTCGAACGACTC	CGTCTGTAGCATCAGGGTACTG	NM-000582
**ALP**	CTGGGCTCCAGGGATAAAGC	TCAGTGTCTCTTGCGCTTGG	NM-000478.4
**OPG**	CCGGAAACAGTGAATCAACTC	AGGTTAGCATGTCCAATGTG	NM-002546.3
**COL1**	AGCGGACGCTAACCCCCTCC	CAGACGGGACAGCACTCGCC	NM-000088.3
**β-Tub**	GAGGGCGAGGACGAGGCTTA	TCTAACAGAGGCAAAACTGAGCACC	NM-001069.2

*OC* osteocalcin, *OP* osteopontin, *ALP* alkaline phosphatase, *OPG* osteoprotegerin, *Col1* collagen type 1α1, *β-Tub* β-tubulin

### Statistical analysis

Results were calculated with GraphPad Prism 6. Samples are not normally distributed; therefore, a non-parametric one-way ANOVA test was performed followed by Tukey’s multiple comparison test. Statistical significance was set for *p* < 0.05.

## Results

### Alkaline phosphatase activity

**Fig. 1:**
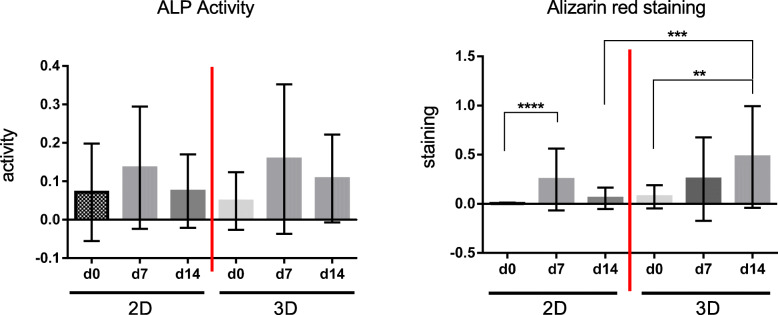
Comparison of ALP activity and alizarin red staining (mineralization) of 2D versus 3D cultures in advanced donor-age osteoblasts after an observation period of 14 days. *p < 0.05, **p < 0.01, ***p < 0.001, ****p < 0.0001

In both cultivation systems, an increase of ALP activity levels was present on day 7 (2D: 1.9-fold, 3D: 3.3-fold) compared to day 0 (Fig. 1). The ALP activity levels were followed by a reduction on day 14 in both cultivation systems compared to day 7. The kinetic course is similar in both groups but with higher levels by trend in the 3D culture.

### Alizarin red staining (mineralization)

On day 7, similar increased levels could be observed in both groups compared to day 0 (2D over 100-fold; *p* < 0.0001) (Fig. 1). A distinct reduction of mineralization was following on day 14 in 2D culture compared to day 7. In the 3D group, a continuous increase was present until day 14 with significant higher levels (6.6-fold) compared to the basic value on day 0 (*p* = 0.0013). Comparing the two culture systems after 14 days, 8.4-fold higher levels were detected in the 3D group (*p* = 0.0002).

### Gene expression

#### Alkaline phosphatase (ALP)

**Fig. 2:**
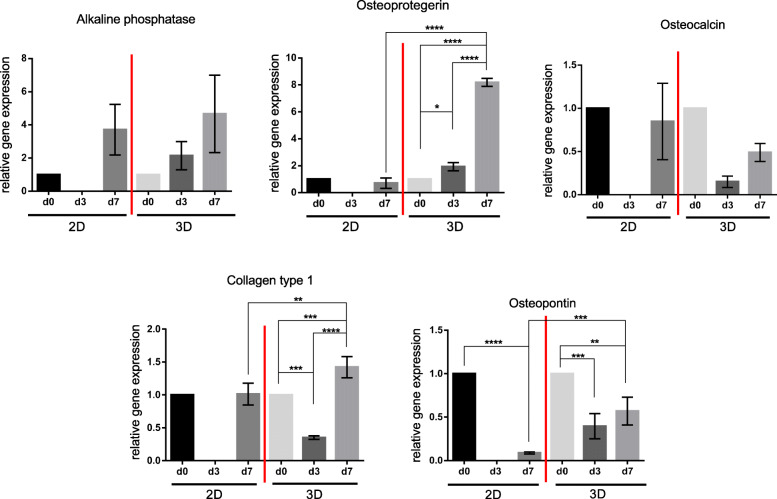
Relative gene expression of ALP, OPG, OC, Col1 and OP. Comparison of 2D versus 3D cultures in advanced donor-age osteoblasts after an observation period of 14 days. *p < 0.05, **p < 0.01, ***p < 0.001, ****p < 0.0001

In the 2D group, a 3.7-fold increase was observed on day 7 compared to day 0, but no statistical difference was present (Fig. 2). At similar basic values, a 4.7-fold gain on day 7 compared to day 0 was demonstrated in the 3D group after a constant increase over the observation period.

#### Osteoprotegerin (OPG)

The 2D group showed a slight reduction of expression levels on day 7 compared to day 0 (Fig. 2). At equal basic levels, the 3D group demonstrated a significant 8.2-fold increase of OPG expression comparing day 0 with day 7 (*p* < 0.0001); day 0 with day 3 (1.9-fold; *p* = 0.0398); and day 3 with day 7 (4.25-fold, *p* < 0.0001) of the 3D culture. Comparing the two cultivation systems, an 11.6-fold increase of OPG levels was present in the 3D group after the observation period of 7 days (*p* < 0.0001).

#### Osteocalcin (OC)

Both groups demonstrated a reduction of OC levels after 7 days compared to day 0 (Fig. 2). In 3D culture, a distinct initial decrease was observed on day 3 compared to day 0. Followed by an increase up to 50% of the basic value of day 0 on day 7. After 7 days, slightly elevated OC levels (85% of the basic value of day 0) compared to the 3D group were found in the 2D group.

#### Collagen type 1α1 (Col1)

Both groups again revealed similar basic values at day 0 (Fig. 2). The 2D osteoblasts showed a steady expression rate of Col1 on day 7. After a significant decline in the 3D group on day 3 (*p* = 0.0004) compared to day 0, a 1.4-fold increase above the basic value (day 0) was observed on day 7 (*p* = 0.009). From day 3 to day 7, a 4-fold rise was present in the 3D group (*p* < 0.0001). Comparing the two cultivation systems, 1.4-fold higher Col1 levels were seen in the 3D group at the end of the observation period (*p* = 0.0077).

#### Osteopontin (OP)

The initial high levels of OP in the 2D culture showed a significant reduction on day 7 compared to day 0 (*p* < 0.0001) (Fig. 2). In the 3D group, there was also a significant reduction of 60% on day 3 (*p* = 0.0001) compared to day 0, followed by a slight increase up to 50% of the basic value of day 0. On day 7, it still represented a significant reduction compared to the levels on day 0 (*p* = 0.0015). Comparing the two cultivation systems, 6.5-fold higher levels of OP were present in the 3D group after 7 days of observation (*p* = 0.0002).

## Discussion

The data in this study indicates that elderly primary human osteoblasts achieve higher osteogenic levels in 3D than in monolayer culture. Col1 was significantly higher in the 3D culture. Col1 is expressed in the early phase of proliferation and indicates an early stage of maturation. This protein is also the most abundant compound in the extracellular matrix (ECM) of bone [28]. The expression of OP and OC are indicators of maturation of ECM [28]. OP levels were significantly higher in 3D culture after the observation time. By trend, levels of OC were slightly lower than in monolayer culture. Alizarin red staining was significantly higher in the 3D cultivated cells representing the mineralization of ECM. The expression of OPG was also significantly elevated in 3D culture. An explanation for these significantly elevated gene expressions of osteogenic markers of elderly primary human osteoblasts in 3D culture might be that osteoblasts are primarily used to a 3D environment. In vivo osteoblasts are primarily positioned in a 3D arrangement. Due to cell isolation via out-migrating in monolayer culture, cells are not able to maintain mature phenotype [29]. After cultivation in monolayer, culture cells were rearranged in a 3D structure mimicking in vivo conditions. This consideration is supported by elevation of early markers (Col1), maturation makers (OP) and mineralization evidence (alizarin red staining), representing improved results during the whole osteogenic process.

Interesting is also the comparison of this present data of old osteoblasts in 3D culture to data of a previous conducted study of ours on old adipose derived mesenchymal stem cells (adMSCs) in 3D culture [30]. The previous study followed the same experimental setup including the same 3D cultivation systems and materials, concentration of osteogenic medium, investigations and observation period with the same time-points as in this present study. The comparison revealed an elevation of early osteogenic markers in osteoblasts. Levels of Col1 were significantly higher (*p* < 0.0001) and ALP was by trend higher in osteoblasts in 3D compared to elderly adMSCs in 3D. OPG levels are also significantly elevated in osteoblasts compared to adMSCs (*p* < 0.0001).

OP, an early marker, signalling the end of proliferation and the beginning of ECM deposition did not show a significant difference comparing the OP expression of elderly osteoblasts and elderly adMSCs in 3D culture. OC, being a late marker of osteoblast maturation in the mineralization phase, did not reveal a significant difference between elderly osteoblasts and elderly adMSCs in 3D culture as well. The alizarin red staining illustrating mineralized ECM was by trend higher in elderly osteoblasts compared to elderly adMSCs in 3D culture. This comparison of data demonstrated elevated early osteogenic markers (Col1, ALP) in 3D osteoblasts. In contrast to adMSCs, osteoblasts might have an advantage, because they do not have to undergo the whole process of differentiation. Furthermore, due to the presence of a 3D environment prior to isolation, osteoblasts are supposedly used to the 3D arrangement. Therefore, this cell type might readjust faster to the 3D environment and resume its function. The fact that OC, as a late osteoblast marker and OP as a further maturation marker, exhibited low levels in both groups might be explained that both cell types were not fully (re-) differentiated yet. However, elderly osteoblasts seem to return faster to their original function than eldery adMSCs, due to the higher expression of early osteogenic markers. Concluding from this data adMSCs should be differentiated into osteoblasts in vitro before being brought in vivo for therapeutic use to achieve higher quality of bone formation.

This study is one of few directly comparing 2D and 3D culture systems [30–32]. A variety of 3D cultivation systems (bioreactors, scaffolds, additives and compounds) makes it difficult to obtain direct comparisons. In this study, the same culture material (polystyrene) without any additives was used for both culture systems. A further positive aspect is that this study is based on a model using primary osteoblasts. A popular choice for osteoblast culture studies are immortalized cell lines, which have the advantage of defined characteristics and a known differentiation state [29, 33, 34]. Still, immortalized cells cannot truly recapitulate the phenotype of a primary osteoblast, and because of this, it is favourable to have a model based on primary osteoblasts as in this present study [29]. A limitation of this study would be missing complete physiological conditions in vivo, which can only be mimicked with a 3D arrangement in vitro.

Summarizing, elderly human primary osteoblasts reveal significantly higher gene expression levels of OPG, Col1 and OP in 3D culture than in 2D culture. Therefore, a relatively high potential of bone formation in a natural 3D arrangement is presumably still present in osteoblasts of advanced donor age (elderly people). Osteoblasts may not be used for therapeutic intention, but this insight is important for physiological understanding. Autologous bone transplantation is in fact the transplantation of osteoblasts in a natural matrix. Therefore, in elderly trauma patients, additional osteogenic measures may be needed. The identification of the mechanisms leading to impaired fracture healing in patients with increased age is of significant importance in order to allow prevention and better management of these healing complications in the elderly.

The clinical relevance of these findings indicate a valid consideration to use osteoblasts of advanced donor age for bone tissue engineering purposes to treat bone defects or mal/non-unions in elderly patients like some other well-established procedures for other tissues like autologous chondrocyte implantation. For basic research, a combination of such osteoblasts of advanced donor age and mesenchymal stem cells in co-culture experiments with a longer observational period would be of interest to evaluate the impact of these findings for bone tissue engineering purposes.

## Data Availability

The datasets used and/or analysed during the current study are available from the corresponding author on reasonable request.

## Abbreviations

2D: Two-dimensional
3D: Three-dimensional
adMSC: Adipose-derived mesenchymal stem cell
ALP: Alkaline phosphatase
BMP: Bone morphogenetic protein
Col1: Collagen type 1α1
ECM: Extracellular matrix
IGF: Insulin-like growth factor
MSC: Mesenchymal stem cell
OC: Osteocalcin
OP: Osteopontin
OPG: Osteoprotegerin
PCR: Polymerase chain reaction
SRB: Sulforhodamine B
